# Identification of Secondary Metabolites from *Aspergillus pachycristatus* by Untargeted UPLC-ESI-HRMS/MS and Genome Mining

**DOI:** 10.3390/molecules25040913

**Published:** 2020-02-18

**Authors:** Bruno Perlatti, Nan Lan, Yongying Jiang, Zhiqiang An, Gerald Bills

**Affiliations:** 1 Texas Therapeutic Institute, The Brown Foundation Institute of Molecular Medicine, University of Texas Health Science Center at Houston, Houston, TX 77054, USA; nan.lan@uth.tmc.edu (N.L.); zhiqiang.an@uth.tmc.edu (Z.A.); gerald.f.bills@uth.tmc.edu (G.B.); 2 Institute for Applied Cancer Science, M.D. Anderson Cancer Center, Houston, TX 77054, USA; yjiang4@mdanderson.org

**Keywords:** *Aspergillus pachycristatus*, metabolic networking, global regulators, LaeA, veA, mcrA

## Abstract

*Aspergillus pachycristatus* is an industrially important fungus for the production of the antifungal echinocandin B and is closely related to model organism *A. nidulans*. Its secondary metabolism is largely unknown except for the production of echinocandin B and sterigmatocystin. We constructed mutants for three genes that regulate secondary metabolism in *A. pachycristatus* NRRL 11440, and evaluated the secondary metabolites produced by wild type and mutants strains. The secondary metabolism was explored by metabolic networking of UPLC-HRMS/MS data. The genes and metabolites of *A. pachycristatus* were compared to those of *A.* *nidulans* FGSC A4 as a reference to identify compounds and link them to their encoding genes. Major differences in chromatographic profiles were observable among the mutants. At least 28 molecules were identified in crude extracts that corresponded to nine characterized gene clusters. Moreover, metabolic networking revealed the presence of a yet unexplored array of secondary metabolites, including several undescribed fellutamides derivatives. Comparative reference to its sister species, *A. nidulans*, was an efficient way to dereplicate known compounds, whereas metabolic networking provided information that allowed prioritization of unknown compounds for further metabolic exploration. The mutation of global regulator genes proved to be a useful tool for expanding the expression of metabolic diversity in *A. pachycristatus*.

## 1. Introduction

Secondary metabolites from fungi are a plentiful and valuable source of molecules that exhibit a wide range of chemical structures and biological activities. Manipulation of culture media composition and genetic mutations can influence the metabolic outcome of culturing a fungus and expand overall numbers of detectable metabolites. However, recognition of effective combinations of parameters that will stimulate the biosynthesis of novel or cryptic metabolites often requires growing a strain in question in many different experiments, leading to cumbersome workflows and repeated encounters of the same compounds [1]. Development and application of efficient methods for molecular dereplication in complex samples with minimal sample work-up are needed to guide the decision-making process. One such method employs untargeted liquid chromatography coupled to tandem high-resolution mass spectrometry (LC-HRMS/MS) and takes advantage of both high-resolution monoisotopic mass measurements and MS/MS fragmentation profiles to generate data which can be used to detect and classify molecules within a single run [2,3]. The significant amounts of data generated are interrogated against databases to identify known substances without the need for purification of each individual component. These spectra can further be used as input for generation of molecular networks, clusters of known and unknown molecules linked based on similarities found in their MS and MS/MS spectra, thus allowing for the classification of a large number of molecules, including previously unidentified substances [4].

*Aspergillus pachycristatus* NRRL 11440 (= ATCC 58397) and its mutants, also previously referred to as *A. nidulans* var. *roseus* and *Emericella rugulosa* [5,6], have been used for industrial-scale production of echinocandin B, a lipohexapeptide used as starting material for the semisynthetic antifungal drug anidulafungin [7,8,9]. A draft genome sequence of *A. pachycristatus* NRRL 11440 revealed a remarkable similarity between *A. pachycristatus* and *A. nidulans*, a model organism that has had its secondary metabolism studied extensively during the past decade. The collection of studies aimed at understanding *A. nidulans* secondary metabolome has led to the discovery of at least 44 gene clusters responsible for the biosynthesis of over 100 secondary metabolites [10,11]. However, only a few secondary metabolites have been described for *A. pachycristatus*, including echinocandins and sterigmatocystin [5,7]. Recently we have determined that the LaeA (*Apc.laeA*) and VeA (*Apc.veA*) global regulatory system was operative in *A. pachycristatus* NRRL 11440 [12]. Disruption of genes encoding these key proteins negatively affected the production of echinocandin B and nearly abolished sterigmatocystin production. We also observed that *Apc.laeA* and *Apc.veA* were essential for normal conidiation and formation of ascomata in *A. pachycristatus*. These experiments focused exclusively on the echinocandin and sterigmatocystin pathways, but we recognized that these mutations also profoundly affected multiple secondary pathways in these mutant strains in a predictable and reproducible manner [12].

We hypothesized that by comparisons to a closely related reference species, viz. *A. nidulans*, as a genetic and metabolic database, we could quickly characterize a portion of the metabolome of *A. pachycristatus* while gaining a measurement of the extent of novelty available for further chemical exploration. Therefore, we aimed to interrogate the secondary metabolism of *A. pachycristatus* NRRL 11440 with previous data from the *A. nidulans* FGSC A4 metabolome and genome as a proxy to characterize its metabolites and tentatively attribute them to their corresponding gene clusters. These analyses provided new insights regarding the degree of divergence in secondary metabolism expected between sibling species of *Aspergillus*. We analyzed extracts from two modified wild-type strains and three mutant strains of NRRL 11440 with disruptions in three global regulator genes known to remodel *Aspergillus* secondary metabolism. These disrupted genes included *laeA* and *veA*, two global regulators of secondary metabolism in *Aspergillus* species [13,14,15,16,17], and *mcrA*, a recently discovered master transcription regulator of secondary metabolism [18].

## 2. Results and Discussion

### 2.1. Metabolic Diversity in Aspergillus pachycristatus

**Figure 1:**
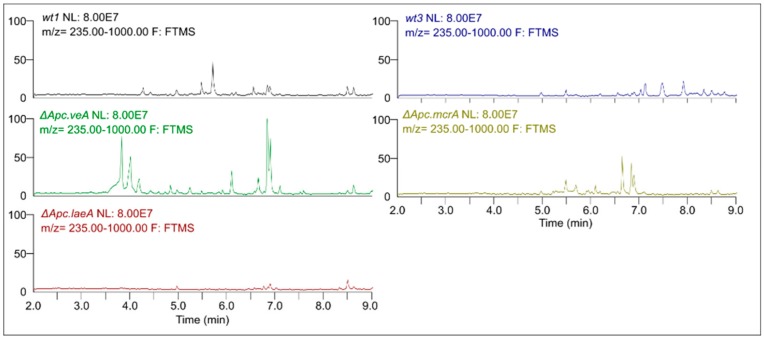
Representative UPLC-HRMS chromatograms obtained for extracts from wt1, △*Apc.laeA*, △*Apc.veA*, wt3, and △*Apc.mcrA* strains of *A. pachycristatus* NRRL 11440 grown in SMY medium.

Five strains of *A. pachycristatus*, designated wt1, △*Apc.laeA*, △*Apc.veA*, wt3, and △*Apc.mcrA* (Figure S1, Table S1) were grown in the same set of culture conditions, and the crude extracts were analyzed by UPLC-HRMS/MS. The resulting chromatograms (Figure 1) were plotted on the same scale corresponding to a maximum peak height to highlight their differences.

Remarkable qualitative differences were evident among them indicating changes in secondary metabolism. It was possible to observe the appearance of several peaks in △*Apc.veA* and △*Apc.mcrA* when compared to their respective parental strains, wt1 and wt3, helping to expand the array of detectable molecules. On the other hand, as we had observed in our previous experiments [12], the △*Apc.laeA* mutant showed remarkable absence of peaks, with almost no detectable signals.

The raw MS data were submitted to the GNPS platform (https://gnps.ucsd.edu; ID=5b9e80c94f054e0d91f194be81594019) in order to build a molecular network containing the detected metabolites of *A. pachycristatus*. A total of 2183 different MS^2^ spectra collected from among the five samples were clustered in 920 nodes according to its MS^2^ fragmentation. The databases in GNPS displayed positive matches for 11 of those nodes. Verification based on identity, HRMS monoisotopic mass deviation and MS^2^ spectra led to the confirmation of three of those secondary metabolites as **4**, **14,** and **21**. The MS^1^ exact masses were compared with known *A. nidulans* secondary metabolites, which allowed the putative identification of 19 additional molecules (**1**–**3**, **5**–**13**, **15**–**20**) (Table 1). MS^2^ spectra were used to validate identified molecules when possible. Echinocandins were not detected by UPLC-HRMS/MS because the mass cutoff was set at *m/z* = 1000, although HPLC-MS found echinocandins in extracts of all strains (Figure S2).

**Figure 2:**
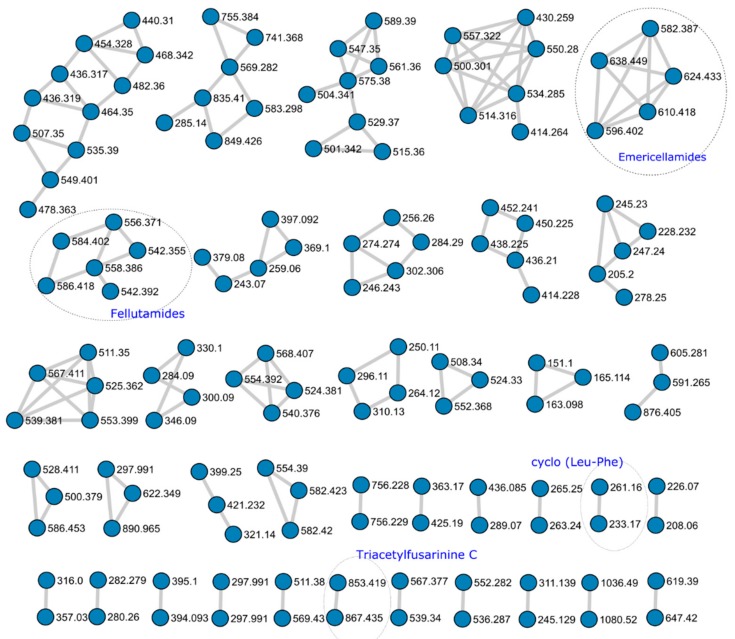
Metabolic Network cluster obtained from the UPLC-HRMS analysis of *Aspergillus pachycristatus* mutants grown in SMY medium showing network clusters with two or more nodes. Numbers represent the HRMS *m/z* observed for each node. Circled clusters indicate molecules positively identified.

From the 920 nodes, the GNPS generated 38 network clusters containing two or more nodes. Of the 136 nodes used, 11 corresponded to molecules identified by GNPS or HRMS (Figure 2).

Two network clusters were further investigated to identify putative unknown nodes based on MS/MS fragment evaluation.

The GNPS database identified the molecule N,N’,N’’-triacetylfusarinine (TAFC) based on MS^1^ and MS^2^ spectra (Figure S3). TAFC is a cyclic molecule composed of three N^5^-*cis*-anhydromevalonyl-N^5^-hydroxy-N^2^-acetyl-L-ornithine residues. *Aspergillus nidulans, A. fumigatus* and other *Aspergillus* species produce it as a major extracellular iron siderophore [19,20]. The TAFC network node was clustered with an unknown nodule showing remarkable similarity in MS^2^ fragmentation (Figure 2). The major difference arose from an increment of *m/z* = 14.0157 Da in MS^1^ adducts, and MS^2^ evaluation indicated the presence of one extra methylene in one of the repeating units of the structure (**22**; Figure S4) The absence of an ion with *m*/*z* = 741.36 suggested the presence of a N^6^-*cis*-anhydromevalonyl-N^6^-hydroxy-N^2^-acetyl-L-lysine Although there are a few examples of bacterial siderophores containing -N^6^-hydroxy-N^2^-acetyl-L-lysine [21,22,23], fungal siderophores have not been reported to incorporate this unit [24,25,26,27]. Further MS^1^ evidence suggested the presence of di- and tri- substituted units, as observed by the MS^1^ spectra of ions *m/z =* 881 (**23**) and *m/z* = 895 (**24**) showing the same clustered adduct ions in MS^1^, but they were not obtained with enough intensity to produce observable MS^2^ spectra (Figure S5). It is possible to infer that the substitution might be observed in any number of the three N^5^-cis-anhydromevalonyl-N^5^-hydroxy-L-ornithine residues, resulting in derivatives of N,N’,N’’-triacetylfusarinine. To the best of our knowledge, derivatives bearing extra methylene units for this class of siderophore have not been reported.

Close inspection of MS^1^ and MS^2^ spectra and comparison with databases and literature enabled the identification of four fellutamide analogs in a constellation of six nodes, antibiotic 1656G (**10**; *m/z* = 584.402) antibiotic 3127 (**11**; *m/z* = 542.355), fellutamide B (**12**; *m/z* = 556.371) and fellutamide C (**13**; *m/z* = 558.386). Fellutamides are tripeptides aldehydes obtained from *Penicillium*, *Aspergillus,* and *Metulocladosporiella* species that inhibit the eukaryotic proteasome [28,29,30,31]. The fellutamide C identified here is the same as identified by Lee and co-workers [28], and not the one characterized from a *Metulocladosporiella* species [31] that was later renamed fellutamide E [32]. 

**Figure 3:**
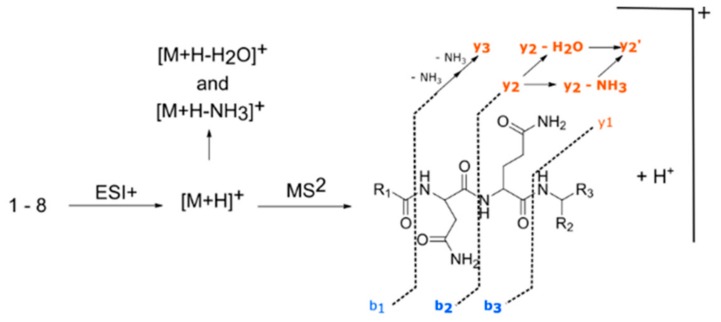
Description of molecular diversity for the different fellutamides observed.

Analysis of MS^2^ spectra of the four fellutamides showed a common fragment associated with peptides fragmentation mechanisms (Figure 3) that allowed us to propose putative structures for the two other unidentified nodes (**25** and **26**). Furthermore, manual curation of chromatograms permitted the identification of two other nodes with similar MS/MS fragmentation patterns (**27** and **28**), even though they were not grouped into the molecular network due to the settings used (Table 2). Their MS^1^ and MS^2^ spectra are shown in Figure S6.

When comparing spectra of fellutamides B and C (**12** and **13**), there was a mass increment of *m/z* = 2 in [M + H]^+^ MS^1^ ion, as well as in MS^2^ ions [M + H − NH_3_]^+^, [M + H − H_2_O]^+^ and “*y*” series of fragments, while the MS^2^ “*b*” series of fragments shows the same nominal masses (Figure S5c,d). This feature confirmed the same structure throughout the alkyl side chain, asparagine and glutamine residues, with a modification at the terminal leucinal residue that was reduced to a leucinol. Comparison of spectra from compounds **10** and **25** (Figure S5a,e) showed the exact same characteristics as observed for the pair of spectra **12** and **13**, from which we deduced the structure of **25** as being the leucinol derivative of **10**, thus identifying it as fellutamide derivative 1. 

Compound **26** has the same nominal mass from **11** (*m*/*z* = 542), although both have different retention times and monoisotopic masses for [M + H]^+^ (**11**, *m*/*z* = 542.3557; **26**, *m*/*z* = 542.3914). HRMS monoisotopic mass indicated that the molecular formula of **26** differed from **11** by having one oxygen atom replaced by incorporation of one carbon and four hydrogens. Evaluation of MS^2^ spectra from **11** and **26** (Figure S5b,f) showed that there was a consistent pattern between them, with a decrease of *m/z* = 16 in the “*b*” series on the MS^2^ and an increase of *m*/z = 16 in “*y*” series of **26**. This difference indicated changes with the same overall *m*/*z* occurring in the alkyl side chain and terminal amino acid residues, with loss of oxygen in the former and acquisition of carbon and hydrogens in the latter. The MS^2^ “*y*” series ions in compound **26** matched the ones observed in compound **13**, indicating the same terminal leucinol residue. HRMS *m*/*z* from “*b*” series MS^2^ ions in **26** indicated the absence of hydroxyl in the alkyl side chain. As such, we identified compound **26** as a novel saturated fatty acid fellutamide derivative 2.

Compound **27** had the same *m*/*z* values for MS^2^ “*y^3^*” fragment as in compounds **10** and **12** indicating the same asn-gln-leucinal residue (Figure S5g). Differences were observed in the MS ion [M + H]^+^, as well as MS^2^ ions [M + H − NH_3_]^+^, [M + H-H_2_O]^+^ and “*b*” series fragments, with *m*/*z* values for **27** showing an increase of *m*/*z* = 14 when compared to **12**, and a decrease of *m*/*z* = 14 when matched to compound **10**. As the difference between **10** and **12** relies on an extra C_2_H_4_ on the alkyl side chain, it is possible to infer that compound **27** possesses an intermediate alkyl side chain between them. The pair of MS^2^ spectra from **27** and **28** (Figure S5g,h) showed the same patterns observed for **12**/**13** and **10**/**25**, indicating that **28** was the leucinol analog of **27**. We, therefore, identified **27** as fellutamide derivative 3, and **28** as fellutamide derivative 4. 

An intense [M + H + H_2_O]^+^ peak in MS^2^ spectra of compounds **13**, **25**, **26,** and **28** was observed and was indicative of a molecular structure that facilitates water loss during gas phase fragmentation, which further corroborates the presence of the reduced leucinol residue. To the best of our knowledge, molecules with structures **25**–**28** have not yet been described from natural sources. Moreover, we note that the MS^2^ spectra alone cannot determine with absolute certainty if alkane portions of these new fellutamides are linear, iso- or anteiso- alkanes.

**Figure 4:**
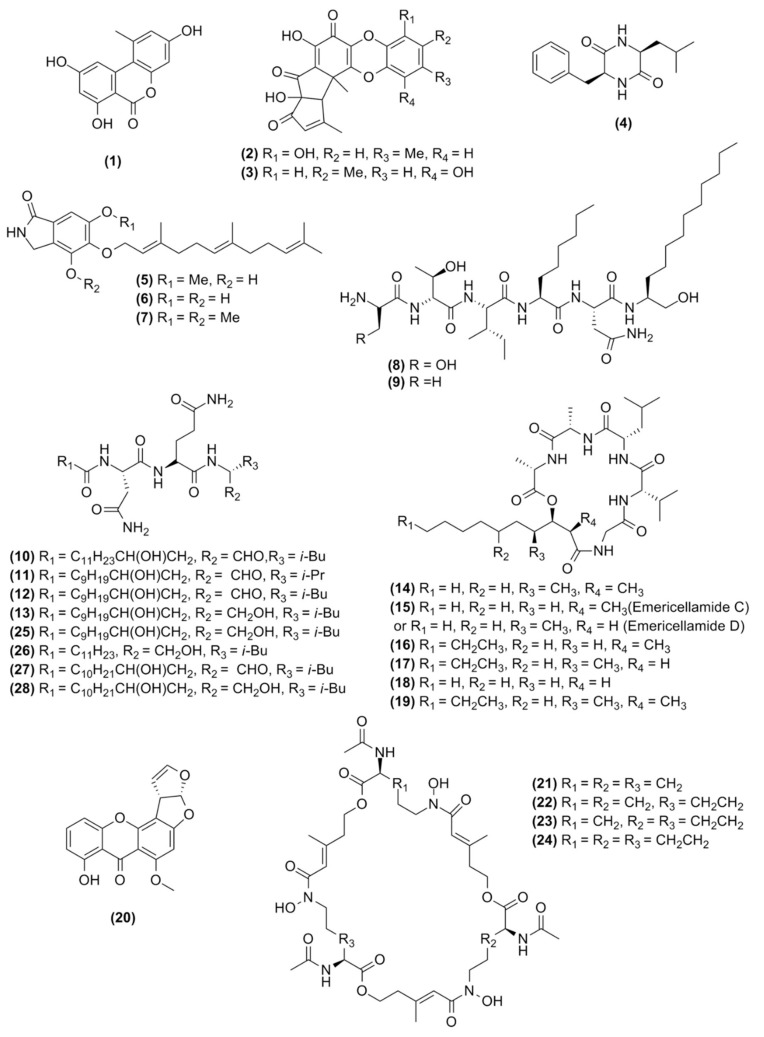
Chemical structure of the molecules observed in this study.

In total, 28 molecules could be putatively identified by combining dereplication strategies using GNPS molecular network, database searches, literature comparisons of HRMS data from *A. nidulans* metabolites, molecular network evaluation, and manual curation of UPLC-HRMS/MS data (Figure 4). 

The overall patterns of secondary metabolite expressed by the different *A. pachycristatus* strains were remarkably different, as evidenced by their chromatograms, but the patterns of metabolite production for the wt1, *ΔApc.laeA* and *ΔApc.veA* strains were remarkably similar to those observed previously [12]. 

We observed various qualitative differences in metabolite expression in the mutants relative to their parent strain controls grown in the same conditions (Figure 1, Table S2). The *laeA* deletion mutant exhibited overall repression of secondary metabolite production that was consistent with our observations for NRRL 11440 [12] and for other *Aspergillus* species [14,16,17,33]. The *veA* mutant showed a more complex metabolite pattern, with repression of several metabolites such as aspercryptins. However, it also showed an increased production of other metabolites such emericellamide, and also the observation of metabolites not detected in wild types such as F9775-A and F-9775-B, a result consistent with previous observations in *A. nidulans* FGSC A4 [15]. For the *mcrA* mutant, a general increase of secondary metabolite production was observed, which parallels previous experiments with the same mutation in *A. nidulans* [18]. 

In addition to the compounds identified above, several compounds observed in *A. pachycristatus* extracts could not be identified by comparison with the *A*. *nidulans* metabolome. Neither could their structures be deduced solely from HRMS and MS/MS experiments. It was evident from the molecular networking (Figure 2) that several of those metabolites were grouped in clusters indicating the production of families of related analogs that could be prioritized for further metabolic studies. These results clearly showed that even though *A. pachycristatus* shares many secondary metabolites with *A. nidulans*, this species produces many more that remain unidentified, of which some are not likely to be produced by *A. nidulans*. In addition, the results from the molecular networking highlight its utility as a dereplication strategy to prioritize unknown metabolites for further evaluation.

### 2.2. Confirmation of Homologous Gene Clusters in A. pachycristatus for the Identified Metabolites

**Figure 5:**
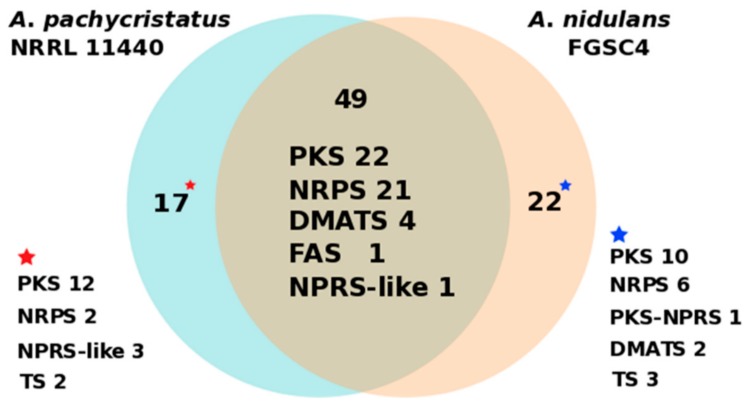
Numbers of distinct and shared secondary metabolite core catalytic genes between A*. nidulans* FGSC4 and *A. pachycristatus* NRRL 11440. PKS (polyketide synthase), NRPS (nonribosomal peptide synthetase), DMATS (dimethylallyltryptophan synthase), FAS (fatty acid synthase), and TS (terpene synthase).

To understand how species-level phylogenetic divergence is reflected in divergence in secondary metabolism, we also compared the overall correspondence of secondary metabolic biosynthetic gene clusters (BGCs) between the strains of *A. pachycristatus* and *A. nidulans* (Figure 5, Table S4). At least 49 core biosynthetic genes showed high amino acid similarities (>63%) between these two strains, of which 22 were PKS, 21 NRPS, 4 DMATS, 1 FAS, and 1 NRPS-like biosynthetic pathways. Furthermore, bioinformatic analysis indicated a capacity for each strain to encode BGCs unique to that strain. *Aspergillus pachycristatus* NRRL 11440 had 17 BGCs exclusive to its genome, while *A. nidulans* had 22. Thus, the two sibling species share approximately two-thirds of their respective secondary metabolite BGCs (Figure 5, Table S4). This degree of similarity was higher than between the sibling species pair of *A. fumigatus* and *A. fishcheri* where 10/33 of *A. fumigatus* BGCs are conserved in *A. fischeri* and only 13/48 *A. fischeri* BGCs are conserved in *A. fumigatus* [34].

Biosynthetic gene clusters in the genome of *A. nidulans* FGSC4 responsible for encoding the enzymes involved in the biosynthesis of targeted molecules were used as templates for searching homologous gene clusters in *A. pachycristatus*. Table 3 shows that for each identified secondary metabolite or metabolite family, the corresponding homologous gene cluster could be found in *A. pachycristatus*. The nonribosomal peptide synthetases (NRPSs) and polyketide synthases (PKSs) from each BGC homolog pair showed remarkably high amino acid similarity (>79%) between these two species (Table 3). Thus, the genetic data confirmed that *A. pachycristatus* genome encodes the biosynthetic capacity for all the identified secondary metabolites. 

Microsynteny of emericellamide, fellutamide, aspercryptin, aspernidine A, orsellinic acid/9775, and sterigmatocystin BGCs was analyzed between *A. pachycristatus* and *A. nidulans* (Figure S7). As expected, all the evaluated BGCs in *A. pachycristatus* consisted of close homologs of the genes encoding these molecules in *A. nidulans*, with a similar cluster organization and gene order which indicated a high degree of certainty that these set of BGCs are responsible for encoding the biosynthesis the metabolites listed in Table 3. 

These BGCs (Table 3) share a high identity and, in all but one instance (Figure S7), and the same gene order between the two strains. However, when we examined the gene cluster boundaries, adjacent genes, and genome loci of these BGCs between *A. pachycristatus* and *A. nidulans*, we unexpectedly found that the corresponding BGCs were located at different genome loci, even though they shared identical boundary genes in almost all cases. Thus, even in close sister species, the secondary metabolic gene clusters are inherited as independent units during the course of evolutionary time. 

## 3. Materials and Methods 

### 3.1. Construction of Gene Deletion and Marker-Recycling Strains

The basic strategy for gene disruption experiments and protoplast transformation methods were previously described in detail [12]. We disrupted the *Apc.pyrG* gene (orotidine-5′-phosphate decarboxylase, MK689400, AN6157 homologue) from NRRL 11440 by protoplast transformation to generate uracil-uridine auxotrophic strains [12]. To facilitate high recombination rates in the modified strain, the DNA double-strand break repair enzyme *Apc.nkuA* (ATP-dependent DNA helicase II, MK689401, AN7753 homolog) was inactivated by insertion of the selective marker pyr4 from *Neurospora crassa*. This strain was designated wt1. Neither substitution of the native *Apc.pyrG* gene with pyr4, or disruption of *Apc.nkuA* appeared to affect colony morphology, sporulation, or secondary metabolism production [12].

For gene disruption, about 1 kb upstream and 1 kb downstream DNA exchange fragments of *Apc*.*stcA* or *Apc*.*mcrA* genes were fused into each end of *afpyrG* cassette by overlap PCR by using PrimeSTAR GXL DNA polymerase. Fusion DNA fragments were purified by QIAquick Gel Extraction Kit before use for protoplast transformation. After protoplast transformation of wt2 (for *stcA* deletion) or wt4 (for *mcrA* deletion) (Table S3) strains, single-colonies were picked up and screened on MMS selection plates without uracil and uridine for at least three generations and then verified by diagnostic PCR (Figures S8 and S10). For *pyrG* marker recycling, about 1 kb upstream and 1 kb downstream DNA exchange fragments of *Apc*.*stcA* gene were cloned from wild type strain and fused together by overlap PCR. Purified fusion DNA fragments were used for protoplast transformation of wt3 strain. Positive transformants were screened on YAS medium supplemented with 1.0 mg/mL 5-FOA, 9 mM uracil and 10 mM uridine and verified by diagnostic PCR (Figure S9). Primers used for gene deletion, marker recycling, and diagnostic PCR are listed in Table S5. Thus, the wt3 strain is identical to wt1 strain, except that *Apc*.*stcA* was inactivated, thus abolishing sterigmatocystin production.

### 3.2. Growth and Sample Preparation

The medium for routine growth and sporulation was yeast extract medium (YAG, 1 L deionized H2O), 5 g yeast extract, 10 g dextrose, 1 mL trace elements (1000 × stock), 2 mL vitamin mix (500 × stock), 10 mL 1 M MgSO4 ·7H2O with or without 1.5% agar. To produce metabolites, strains were grown in 250-mL flasks containing 50 mL SMY medium (Bacto neopeptone 10 g, maltose 40 g, yeast extract 10 g per 1000 ml of deionized H_2_O). Cultures were maintained for 10 days at 24 °C and 220 rpm. For the extraction procedure, an equal volume of ethyl acetate was added to the culture and the extraction carried out overnight, with agitation. The extract was vacuum-filtered, and the organic phase separated. A 5.0-mL aliquot was placed in a clean glass flask and evaporated to dryness at 37 °C under gentle airflow. The dried extract was reconstituted in 0.25 mL of acetonitrile, diluted with 0.25 mL of ultra-pure water, filtered through 0.22 µm RC membrane and submitted to chromatographic analyses.

### 3.3. HPLC-MS Analysis

HPLC-MS data were acquired on an Agilent 1260 HPLC equipped with a diode array detector (DAD) and coupled to an Agilent 6120 single quadrupole mass spectrometer (MS), with an Ace Equivalence 5 C_18_, 4.6 × 150 mm, 5 μm column kept at 30 °C. The pump used a binary solvent system consisting of 0.1% aqueous formic acid (solvent A) and, 0.1% formic acid in acetonitrile (ACN) (solvent B) with the following gradient: 20%−100% B for 22 min, and maintaining 100%B for 4 min, 1.0 mL.min^−1^. The chromatographic profiles were monitored by wavelength scanning from 190 to 400 nm and by positive and negative ESI-MS from *m*/*z* 160–1500. The injection volume was 10 µL.

### 3.4. UPLC-HRMS Analysis

Extracts were analyzed with a Waters Acquity I-Class UPLC with DAD detector and equipped with an Acquity UPLC BEH C18 (1.7 um, 2.1 × 50 mm) at 40 °C, with flow rate of 0.5 mL.min^−1^. The following gradient was applied using 0.1% aqueous acetic acid (solvent A) and 0.1% acetic acid in ACN (solvent B): at 0 min., 5% B, maintained for 2 min, 14 min, 95% B. The column eluent was directed to a Thermo Orbitrap mass spectrometer, operating in the 100–1000 mass range, using as heat temp 375 °C, sheath gas flow rate 45, Aux Gas Flow Rate 10, Sweep Gas Flow Rate 3, I Spray Voltage 4.10 kV, Capillary Temp 320 °C, S-Lens RF Level 55%. The injection volume was 2 µL.

### 3.5. Metabolic Profiling, Molecular Networking and Compound Dereplication

Chromatograms were analyzed with Thermo Xcalibur software for the identification of peaks, measurements of individual peak height, and accurate MS^1^ *m*/*z*. 

Raw data from UPLC–HRMS/MS were converted to mzXML using ProteoWizard (version 3.0.10051, Vanderbilt University, USA). All mzXML data were uploaded to the Global Natural Products Social Networking (GNPS) website (https://gnps.ucsd.edu/ProteoSAFe/static/gnps-splash.jsp). A molecular network was created using the online workflow at the GNPS. The data were filtered by removing all MS/MS peak\s within +/− 17 Da of the precursor *m/z*. MS/MS spectra were window filtered by choosing only the top six peaks in the +/− 50 Da window throughout the spectrum. The data was then clustered with MS-Cluster with a parent mass tolerance of 0.02 Da and a MS/MS fragment ion tolerance of 0.02 Da to create consensus spectra. Furthermore, consensus spectra that contained less than one spectra were discarded. A network was then created where edges were filtered to have a cosine score above 0.7 and more than four matching peaks. Edges between two nodes were kept in the network if, and only if, each of the nodes appeared in each other’s respective top 10 most similar nodes. The spectra in the network were then searched against the GNPS spectral libraries. The library spectra were filtered in the same manner as the input data. All matches kept between network spectra and library spectra were required to have a score above 0.7 and at least six matched peaks. Analog searching was enabled against the library with a maximum mass shift of 0.02 Da [4]. The metabolic network was built using Cytoscape 3.7.0 [35]. The job ID is ID = 5b9e80c94f054e0d91f194be81594019.

Dereplication of known molecules was enabled by matching to hits in GNPS database and aided by manually searching other databases such as the Natural Product Atlas (www.npatlas.org/) and the Dictionary of Natural Products (dnp.chemnetbase.com/). Moreover, due to the genetic similarities of *A. pachycristatus* and *A. nidulans* secondary metabolism, previous works describing *A. nidulans* metabolites were used as proxy set [28,31,36,37,38,39,40,41,42,43]. 

### 3.6. Comparison of Secondary Metabolic Biosynthestic Gene Clusters of A. pachycristatus NRRL 11440 and A. nidulans FGSC A4

The genetically encoded BGCs of *A*. *pachycristatus* NRRL 11440 were predicted by submitting the unannotated scaffold sequences for antiSMASH analysis (https://fungismash.secondarymetabolites.org/) [44,45] and by reciprocal BLAST searches of predicted proteins and annotated scaffolds with sequences of previously determined core genes and gene clusters from *A*. *nidulans* [37,38,42,43,46,47,48] followed by manual correction of some incorrectly predicted ORFs. Orthologous biosynthetic gene clusters between *A*. *pachycristatus* NRRL 11440 and *A*. *nidulans* FGSC A4 were aligned and illustrated using Easyfig [49] to determine gene identity (%) and microsynteny.

## 4. Conclusions

By referencing an extensively explored and annotated model organism as a proxy, we were able to efficiently dereplicate several gene clusters and classes of previously undescribed metabolites from mutant strains of *A. pachycristatus*. Collectively, the results of this study confirm that *A. pachycristatus* shares roughly two-thirds of secondary biosynthetic gene clusters and metabolites with that of *A. nidulans*. However, it still produces or has the potential to produce a large set of yet unknown secondary metabolites and biosynthetic genes that can be further explored as a potential source for the discovery of novel molecules. Moreover, we confirmed the possibility of expanding the observed metabolome of *A. pachycristatus* through the disruption of global regulator genes. The metabolic effects of gene disruptions in *A. pachycristataus* (*laeA, veA, mcrA*) approximately paralleled reported effects in *A. nidulans*. These results provide additional evidence that these gene disruptions can be a useful tool to enhance and vary overall secondary metabolite expression and enabling the discovery of silent and cryptic metabolites. 

## Figures and Tables

**Table 1:** Secondary metabolites identified from *Aspergillus pachycristatus* NRRL 11440.

N°	Molecule	R. Time	Exact Mass [M + H]^+^	*m/z* Found	Error (ppm)	Class
**1**	Alternariol ^b^	4.08	259.0607	259.0603	−1.5	Polyketide
**2**	F9775-A ^b^	3.40	397.0923	397.0921	−0.5	Polyketide
**3**	F9775-B ^b^	3.91	397.0923	397.0920	−0.8	Polyketide
**4**	Cyclo (Leu-Phe) ^a^	3.89	261.1603	261.1600	−1.2	Diketopiperazine
**5**	Aspernidine A ^b^	7.25	400.2488	400.2487	−0.3	Prenylated alkaloid
**6**	Aspernidine B ^b^	6.70	386.2331	386.2324	−1.8	Prenylated alkaloid
**7**	Aspernidine C ^b^	7.29	414.2644	414.2638	−1.5	Prenylated alkaloid
**8**	Aspercryptin A1 ^b^	5.54	758.5392	758.5383	−1.2	Lipopeptide
**9**	Aspercryptin A2 ^b^	5.60	742.5443	742.5432	−1.5	Lipopeptide
**10**	Antibiotic 1656G ^b^	6.40	584.4018	584.4023	0.9	Lipopeptide
**11**	Antibiotic 3127 ^b^	5.58	542.3548	542.3557	1.6	Lipopeptide
**12**	Fellutamide B ^b^	5.68	556.3705	556.3706	0.2	Lipopeptide
**13**	Fellutamide C ^b^	5.47	558.3861	558.3860	−0.2	Lipopeptide
**14**	Emericellamide A ^a^	6.64	610.4174	610.4177	0.5	Cyclic lipopeptide
**15**	Emericellamide C/D ^b,c^	6.09	596.4018	596.4022	0.7	Cyclic lipopeptide
**16**	Emericellamide E ^b^	6.82	624.4331	624.4327	−0.6	Cyclic lipopeptide
**17**	Emericellamide F ^b^	6.89	624.4331	624.4332	0.2	Cyclic lipopeptide
**18**	Emericellamide G ^b^	5.90	582.3861	582.3862	0.2	Cyclic lipopeptide
**19**	Emericellamide H ^b^	7.08	638.4487	638.4488	0.2	Cyclic lipopeptide
**20**	Sterigmatocystin ^b^	5.76	325.0712	325.0706	−1.9	Polyketide
**21**	N,N’,N’’-triacetylfusarinine ^a^	3.82	853.4189	853.4197	0.9	Hydroxamate siderophore

^a^ Compounds identified using GNPS database. ^b^ Compounds identified by matching HRMS with known *Aspergillus nidulans* secondary metabolites. ^c^ It is not possible to distinguish between the two isomers in this case.

**Table 2:** Fellutamide derivatives identified from *Aspergillus pachycristatus* NRRL 11440.

N°	Name	Molecular Formula	[M + H]^+^ Ion	R1	R2	R3
**10**	Antibiotic 1656G	C_29_H_53_N_5_O_7_	584.4023	C_11_H_23_CH(OH)CH_2_	CHO	*i*-Bu
**11**	Antibiotic 3127	C_26_H_49_N_5_O_7_	542.3557	C_9_H_19_CH(OH)CH_2_	CHO	*i*-Pr
**12**	Fellutamide B	C_27_H_49_N_5_O_7_	556.3706	C_9_H_19_CH(OH)CH_2_	CHO	*i*-Bu
**13**	Fellutamide C	C_27_H_51_N_5_O_7_	558.3860	C_9_H_19_CH(OH)CH_2_	CH2OH	*i*-Bu
**25**	Fellutamide derivative 1	C_29_H_55_N_5_O_7_	586.4175	C_11_H_23_CH(OH)CH_2_	CH2OH	*i*-Bu
**26**	Fellutamide derivative 2	C_27_H_53_N_5_O_6_	542.3914	C_11_H_23_	CH2OH	*i*-Bu
**27**	Fellutamide derivative 3	C_28_H_51_N_5_O_7_	570.3862	C_10_H_21_CH(OH)CH_2_	CHO	*i*-Bu
**28**	Fellutamide derivative 4	C_28_H_53_N_5_O_7_	572.4018	C_10_H_21_CH(OH)CH_2_	CH2OH	*i*-Bu

**Table 3:** Core biosynthetic genes from inferred biosynthetic clusters for metabolites identified in *Aspergillus pachycristatus* and their homologs in *A. nidulans.*

Gene in *A. pachycristatus* NRRL 11440	Gene Name *A. Nidulans*	Corresponding Gene FGSC A4	Product	Type	Similarity %
g7715	*atnA*	AN7884	Aspercryptin	NRPS	92.2
g5866	*easA*	AN2545	Emericellamide	NRPS	91.1
g5867	*easB*	AN2547	Emericellamide	PKS	93.7
g7876	*inpA*	AN3495	Fellutamide	NRPS	91.3
g7875	*inpB*	AN3496	Fellutamide	NRPS	92.2
g4770	*sidD*	AN6236	N’,N’’,N’’’-triacetylfusarinine C	NRPS	95.6
g8711	*orsA*	AN7909	Orsellinic acid/F9775	PKS	93.2
g4218	*pkfA*	AN3230	Aspernidine	PKS	94.9
g5606	*pkgA*	AN7071	Alternariol	PKS	79.2
g7351	*stcA*	AN7825	Sterigmatocystin	PKS	93.9

## Supplementary Materials

The following are available online at https://www.mdpi.com/1420-3049/25/4/913/s1, Figure S1. Morphological variations in *Aspergillus pachycristatus* mutants grown on agar media. Figure S2. Extracted ion chromatogram (EIC^+^ = *m*/*z* 1042–1044) from LC-MS analysis of *Aspergillus pachycristatus* strains in SYM media highlighting the production of echinocandin B [M + H − H_2_O]^+^ ion in all tested media. Figure S3. A) MS^1^; B) MS^2^ and C) Proposed fragmentation pathway of N,N’,N’’-Triacetylfusarinine C. Figure S4. A) MS^1^; B) MS^2^ and C) Proposed fragmentation pathway of N,N’,N’’-Triacetylfusarinine C analog containing an extra methylene. Blue fragments indicates *m*/*z* similar to those observed in TAFC, while orange *m/z* represent fragments containing an increased 14 Da. Figure S5. Chromatogram and MS^1^ spectra showing other possible analogs of N,N’,N’’-Triacetylfusarinine C (22, 23) containing extra methylene units, when compared to N,N’,N’’-Triacetylfusarinine C (21) and their putative structure. Figure S6. MS^1^ and MS^2^ HRMS spectra of fellutamides: (a) Antibiotic 1656G (10); (b) Antibiotic 3127 (11); (c) Fellutamide B (12); (d) Fellutamide C (13); (e) Fellutamide derivative 1 (25); (f) Fellutamide derivative 2 (26); (g) Fellutamide derivative 3 (27); (h) Fellutamide derivative 4 (28). Figure S7. Microsynteny comparisons of emericellamide, fellutamide, aspercryptin, aspernidine A, orsellinic acid/9775 and sterigmatocystin BGCs between A*. pachycristatus* NRRL 11440 and *A. nidulans* FGSC A4. Figure S8. Construction of DNA fusion fragments for *apc*.*stcA* deletion and diagnostic PCR. A. Schematic diagram of *Apc.stcA* disruption by insertion of *Af.pyrG* as selective marker gene by homologous recombination. B. Agarose gel images of PCR products of construction of DNA fusion fragments for protoplast transformation. C. Schematic diagram of verification of △*Apc*.*stcA* transformants. D. Agarose gel images of PCR products for detection of *Apc*.*stcA* deletion of eight independent transformants. Figure S9. Recycling of *Af*.*pyrG* marker gene from △*Apc*.*stcA* mutant. A. Schematic diagram of *Af.pyrG* deletion from *Apc*.*stcA* locus and verification of *Af.pyrG* recycling transformants. B. Agarose gel images of PCR products of construction of DNA fusion fragments for protoplast transformation and PCR products for detection of *Af.pyrG* deletion of 10 independent transformants Figure S10. Construction of DNA fusion fragments for *ΔApc.mcrA* deletion and diagnostic PCR. A. Schematic diagram of *Apc.mcrA* disruption by insertion of *Af.pyrG* as selective marker gene by homologous recombination. B. Agarose gel images of PCR products of construction of DNA fusion fragments for protoplast transformation. C. Schematic diagram of verification of △*Apc.mcrA* transformants. D. Agarose gel images of PCR products for detection of *Apc.mcrA* deletion transformants. Table S1. HRMS error for diagnostic MS and MS^2^ ions for fellutamides. Table S2. Qualitative evaluation of the presence of detected metabolites in the extracts of *A. pachycristatus* strains. Table S4. Correspondence of secondary metabolic BGCs between strains *A. pachycristatus* NRRL 11440 and *A. nidulans* FGSC A4. Table S5. Primers used in this study.
