# Supplementary material for: Identification of Secondary Metabolites from Aspergillus pachycristatus by Untargeted UPLC-ESI-HRMS/MS and Genome Mining
Source: Molecules. 2020 Feb 18;25(4):913. doi: 10.3390/molecules25040913 (PMC7071103; doi:10.3390/molecules25040913)
Supplement: Supplementary file 1 [file molecules-25-00913-s001.pdf]

molecules

SUPPLEMENTARY MATERIAL

Untargeted UPLC-HRMS/MS metabolome analysis of *Aspergillus pachycristatus*

Bruno Perlatti [1, †, *], Nan Lan[1, †], Yongying Jiang [2], Zhiqiang An[1] and Gerald F. Bills [1]

[1] Texas Therapeutic Institute, The Brown Foundation Institute of Molecular Medicine, University of Texas Health Science Center at Houston, Houston, Texas 77054, United States; bruno.perlatti@uth.tmc.edu (B.P.); nan.lan@uth.tmc.edu (N.L.); zhiqiang.an@uth.tmc.edu (Z.A.); gerald.f.bills@uth.tmc.edu (G.B.).

[2] Institute for Applied Cancer Science, M.D. Anderson Cancer Center, Houston, TX, USA.; yjiang4@mdanderson.org

* Correspondence: bruno.perlatti@uth.tmc.edu;

[†]These authors contributed equally to this work.

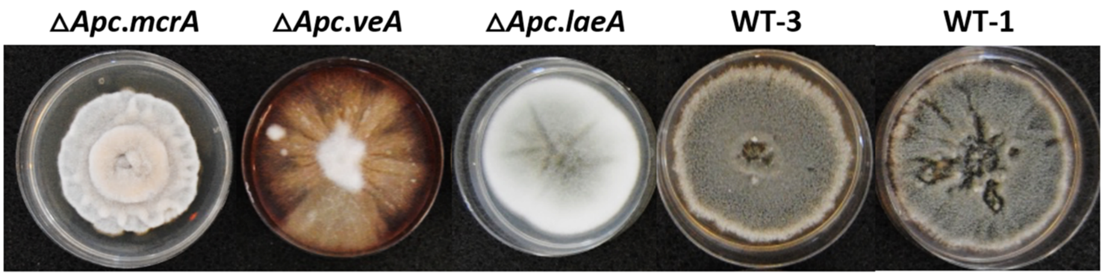

Figure S1. Morphological variations in *Aspergillus pachycristatus* mutants grown on YAG agar media.

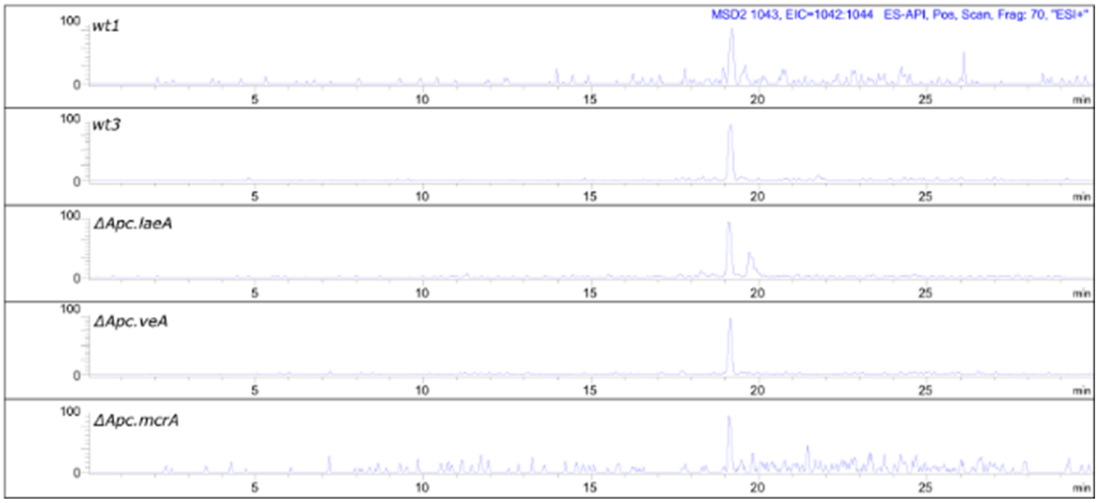

Figure S2. Extracted ion chromatogram (EIC$^+$ = *m*/*z* 1042-1044) from LC-MS analysis of *Aspergillus pachycristatus* strains in SYM media highlighting the production of echinocandin B [M+H-H_2O]$^+$ ion in strains used.

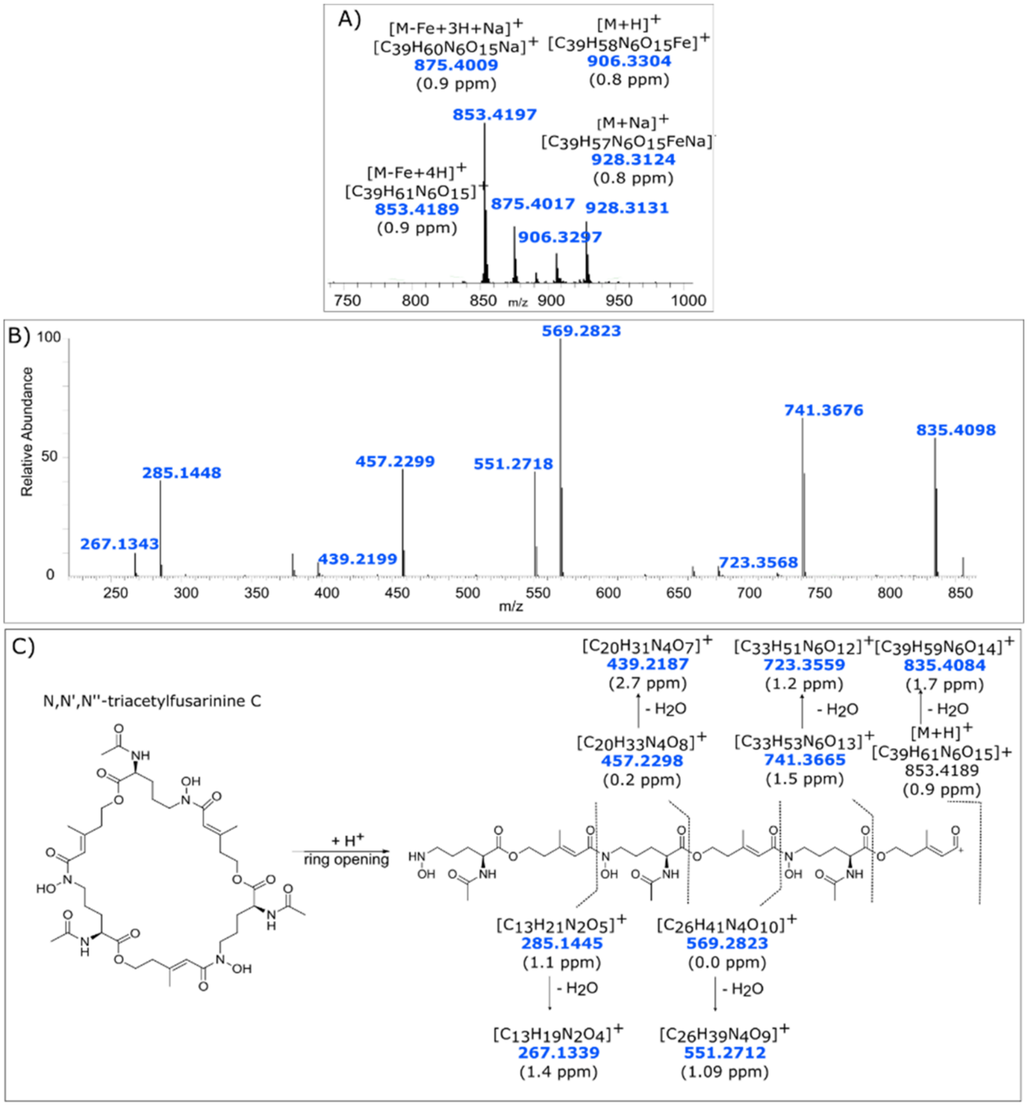

Figure S3. A) MS[1]; B) MS[2] and C) Proposed fragmentation pathway of N,N′,N′′-Triacetylfusarinine C.

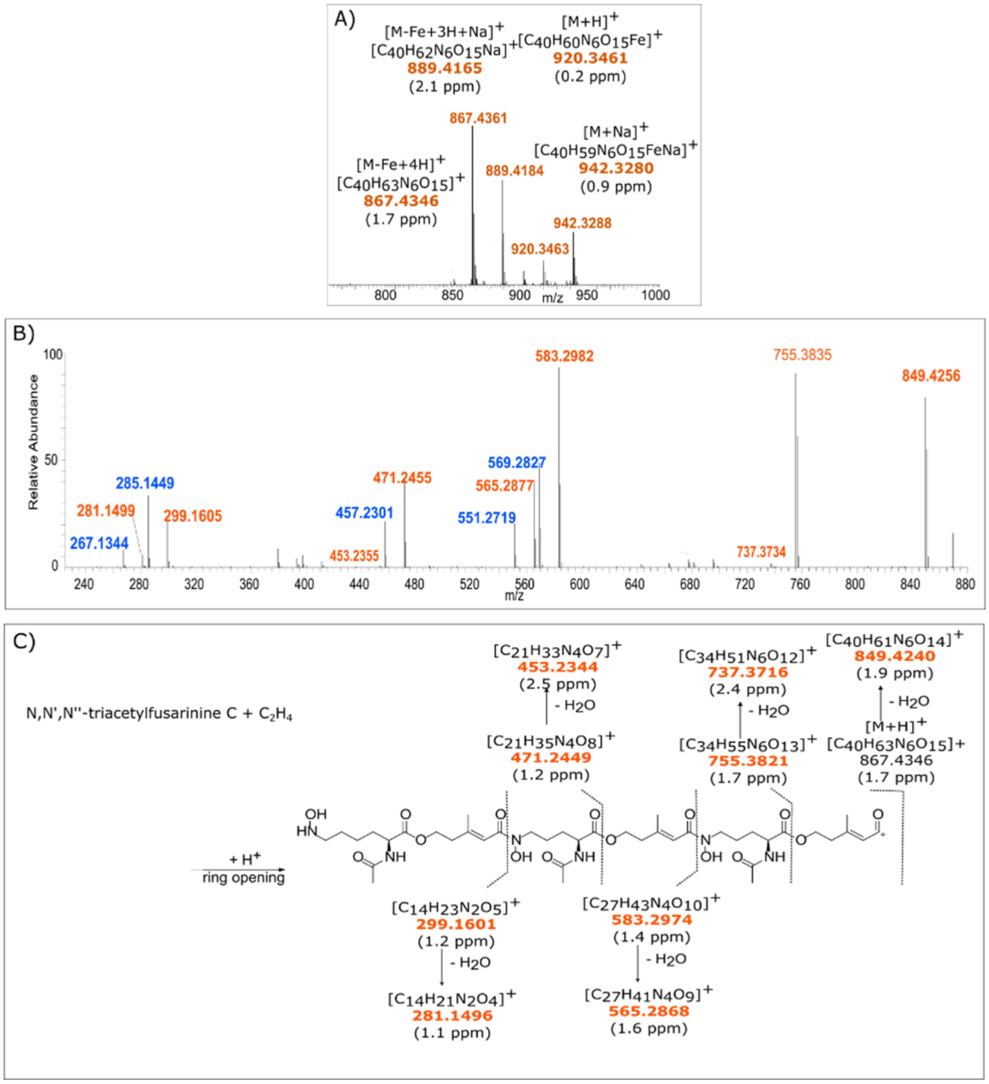

Figure S4. A) MS[1]; B) MS[2] and C) Proposed fragmentation pathway of N,N′,N′′-Triacetylfusarinine C analog containing an extra methylene. Blue fragments indicates *m*/*z* similar to those observed in TAFC, while orange *m*/*z* represent fragments containing an increased 14 Da.

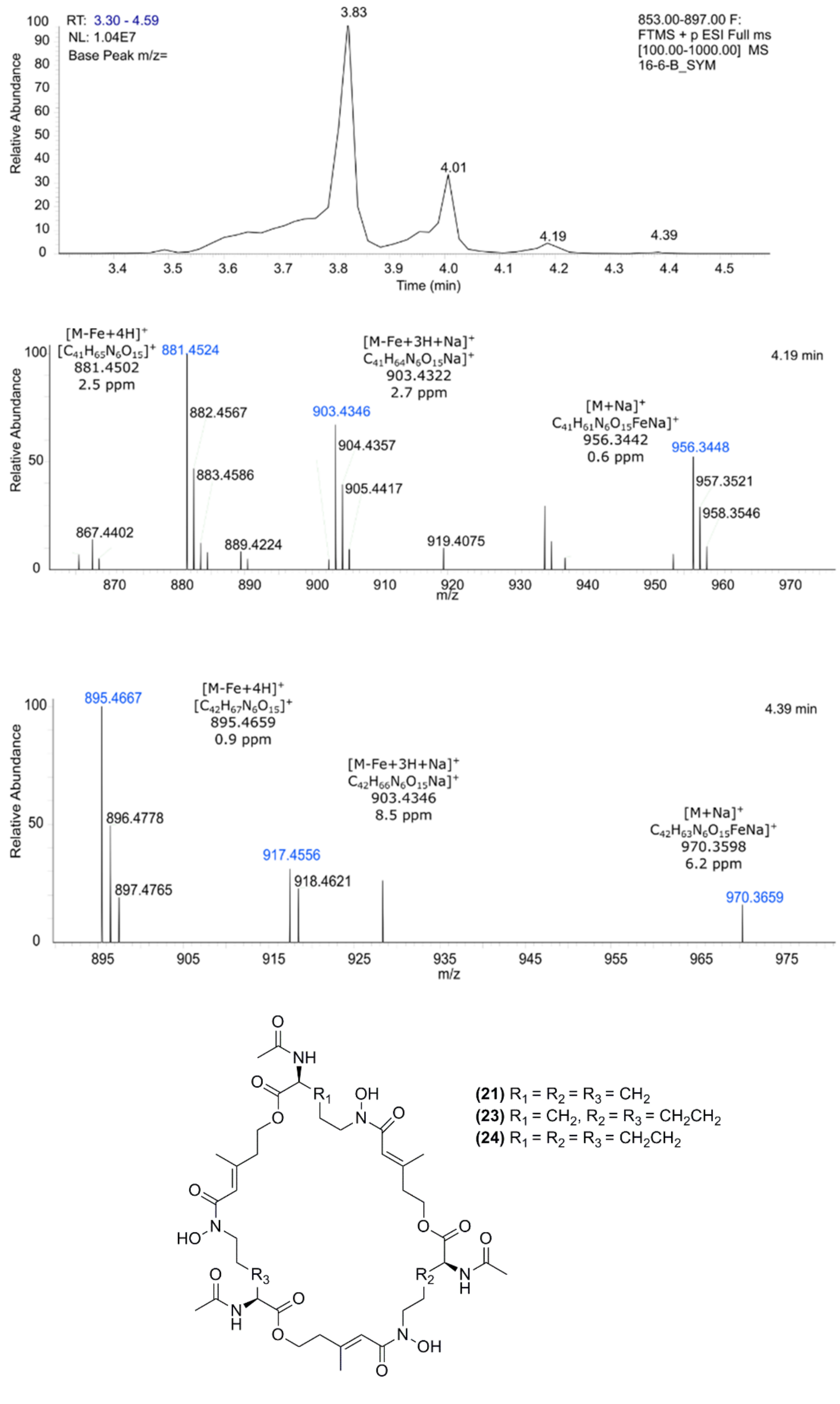

Figure S5. Chromatogram and MS[1] spectra showing other possible analogs of N,N′,N′′-Triacetylfusarinine C (**22**, **23**) containing extra methylene units, when compared to N,N′,N′′-Triacetylfusarinine C (**21**) and their putative structure.

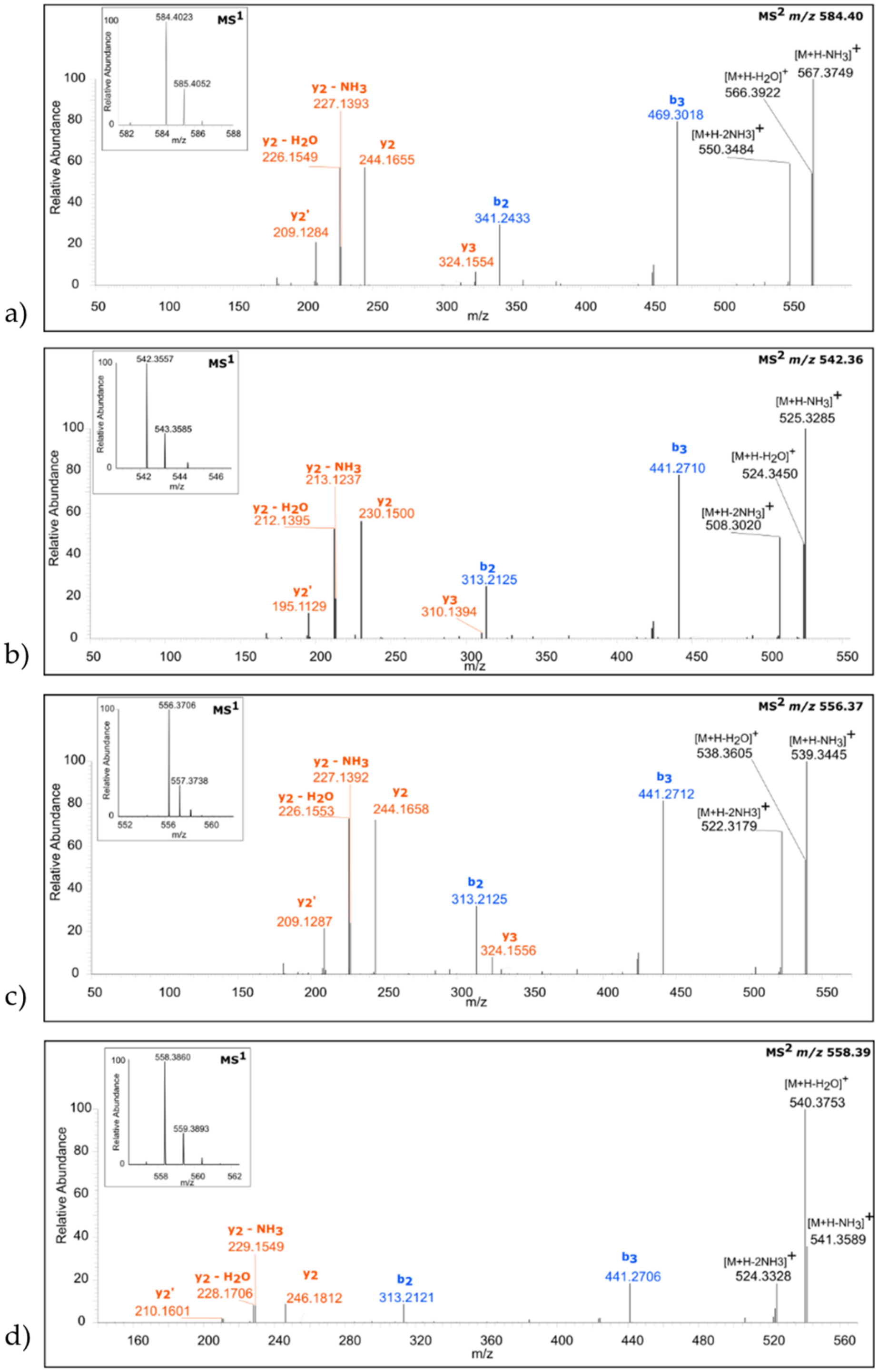

Figure S6. MS[1] and MS[2] HRMS spectra of fellutamides: a) Antibiotic 1656G (10); b) Antibiotic 3127 (11); c) Fellutamide B (12); d) Fellutamide C (13); e) Fellutamide derivative 1 (25); f) Fellutamide derivative 2 (26); g) Fellutamide derivative 3 (27); h) Fellutamide derivative 4 (28).

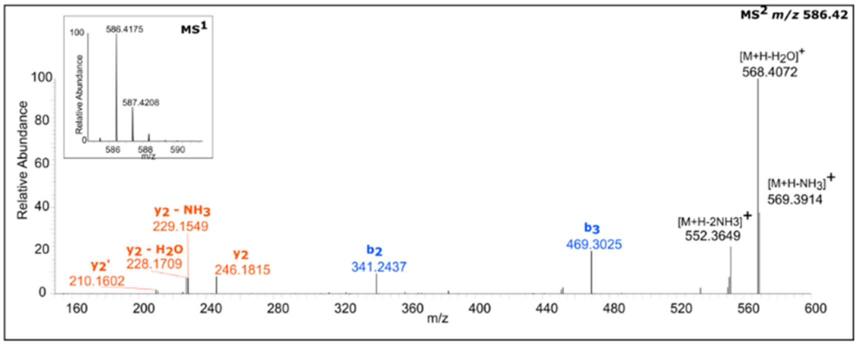

e)

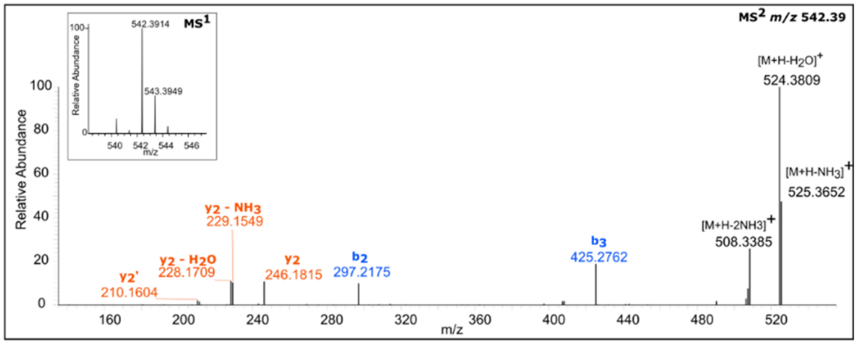

f)

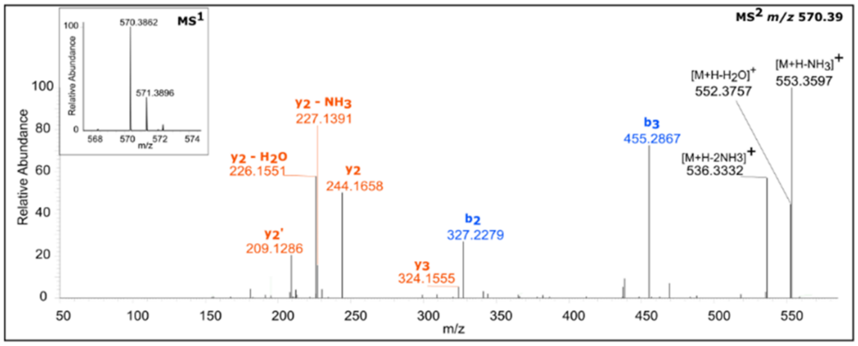

g)

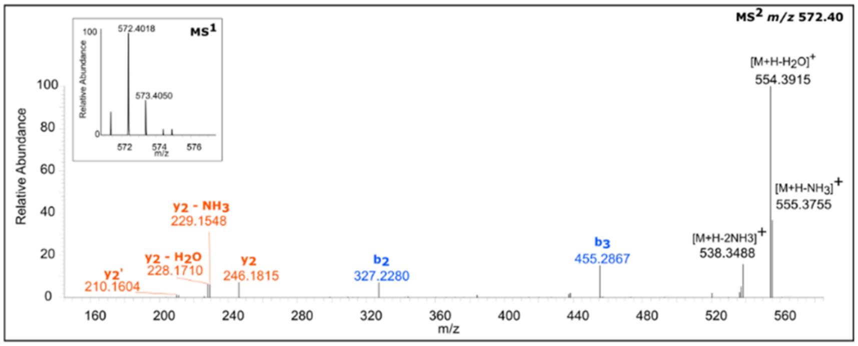

h)

Figure S6. MS[1] and MS[2] HRMS spectra of fellutamides: a) Antibiotic 1656G (10); b) Antibiotic 3127 (11); c) Fellutamide B (12); d) Fellutamide C (13); e) Fellutamide derivative 1 (25); f) Fellutamide derivative 2 (26); g) Fellutamide derivative 3 (27); h) Fellutamide derivative 4 (28).

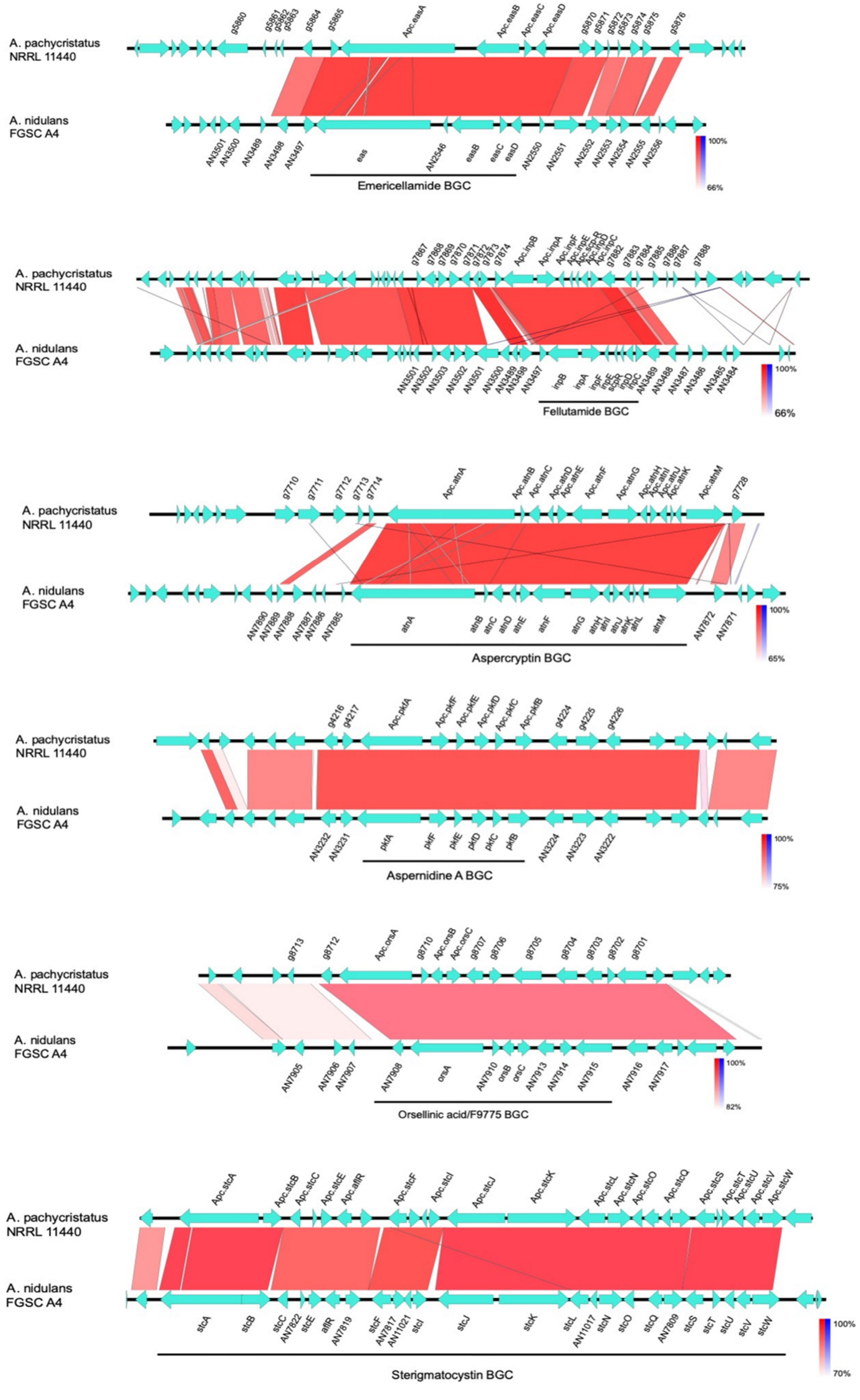

Figure S7. Microsynteny comparisons of emericellamide, fellutamide, aspercryptin, aspernidine A, orsellinic acid/9775 and sterigmatocystin BGCs between A. *pachycristatus* NRRL 11440 and *A. nidulans* FGSC A4.

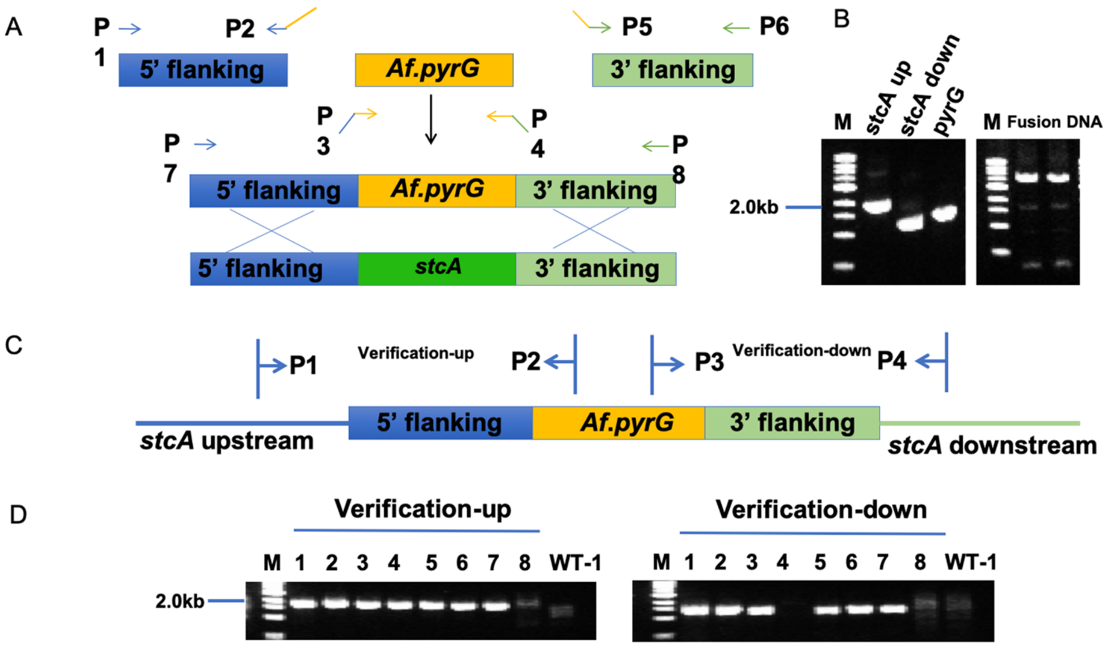

Figure S8. Construction of DNA fusion fragments for *apc.stcA* deletion and diagnostic PCR. A. Schematic diagram of *Apc.stcA* disruption by insertion of *Af.pyrG* as selective marker gene by homologous recombination. B. Agarose gel images of PCR products of construction of DNA fusion fragments for protoplast transformation. C. Schematic diagram of verification of △*Apc.stcA* transformants. D. Agarose gel images of PCR products for detection of *Apc.stcA* deletion of 8 independent transformants.

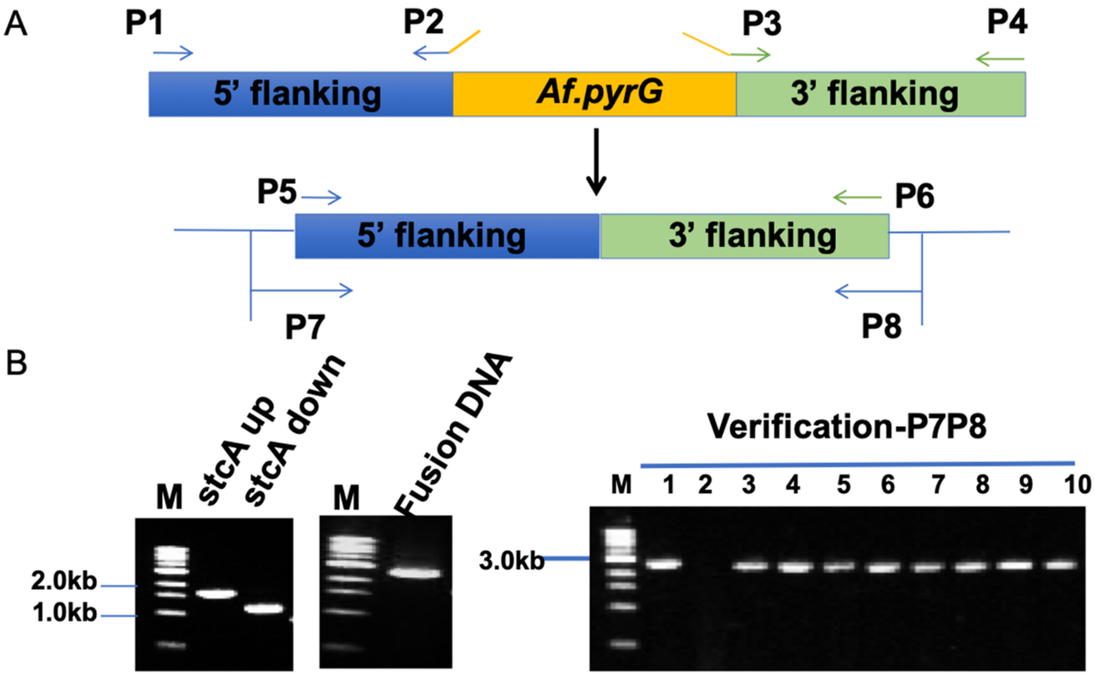

Figure S9. Recycling of *Af.pyrG* marker gene from △*Apc.stcA* mutant. A. Schematic diagram of *Af.pyrG* deletion from *Apc.stcA* locus and verification of *Af.pyrG* recycling transformants. B. Agarose gel images of PCR products of construction of DNA fusion fragments for protoplast transformation and PCR products for detection of *Af.pyrG* deletion of 10 independent transformants.

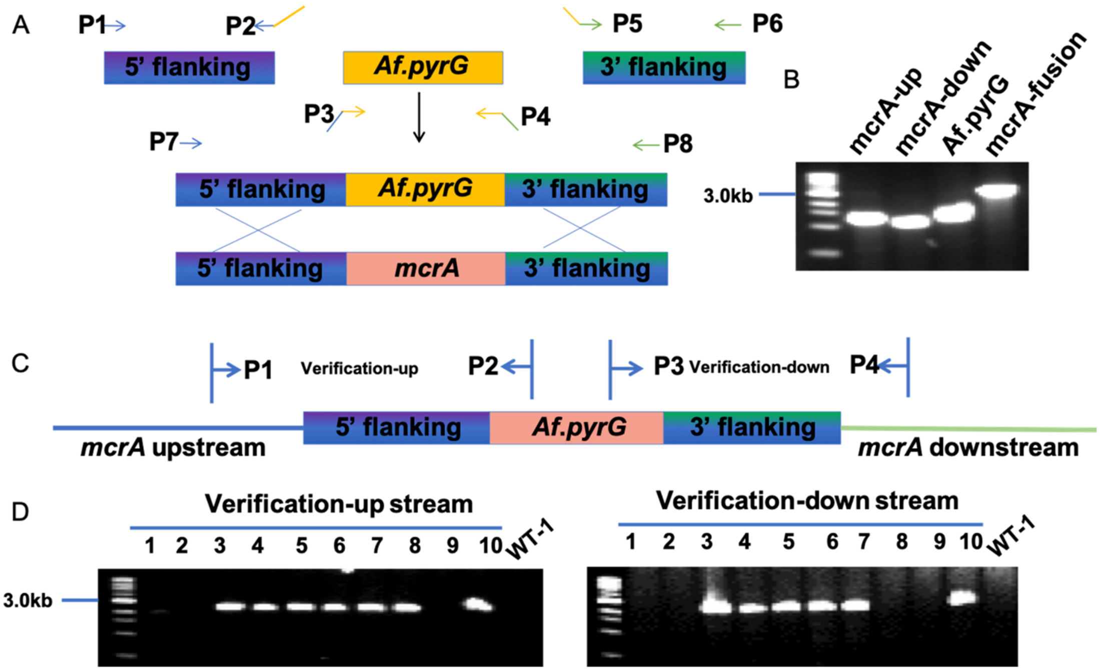

Figure S10. Construction of DNA fusion fragments for *ΔApc.mcrA* deletion and diagnostic PCR. A. Schematic diagram of *Apc.mcrA* disruption by insertion of *Af.pyrG* as selective marker gene by homologous recombination. B. Agarose gel images of PCR products of construction of DNA fusion fragments for protoplast transformation. C. Schematic diagram of verification of Δ*Apc.mcrA* transformants. D. Agarose gel images of PCR products for detection of *Apc.mcrA* deletion transformants.

molecules

Table S1. HRMS error for diagnostic MS and MS^2 ions for fellutamides.

Compound	*10*	*11*	*12*	*13*	*25*	*26*	*27*	*28*
Name	Antibiotic 1656G	Antibiotic 3127	Fellutamide B	Fellutamide C	Fellutamide *derivative 1*	Fellutamide *derivative 2*	Fellutamide *derivative 3*	Fellutamide *derivative 4*
$[M+H]^+$	0.90	1.61	0.22	-0.22	0.13	0.35	0.13	0.04
$[M+H-NH_3]^+$	-0.51	0.41	1.03	-1.16	0.85	0.97	0.19	0.48
$[M+H-H_2O]^+$	-0.50	0.65	1.04	-0.46	0.65	0.52	1.98	0.49
$[M+H-2NH_3]^+$	-0.41	0.48	0.94	-0.40	0.98	0.82	0.30	0.27
b3	-0.45	0.54	1.02	-0.30	0.98	0.85	0.66	1.07
b2	-0.39	1.07	0.98	-0.17	0.75	0.88	0.23	0.60
y3	-0.10	1.75	0.56	-	-	-	1.30	-
y2	-0.28	0.53	1.16	-0.03	1.10	1.02	0.79	0.98
$y2-NH_3$	0.09	0.62	1.28	-0.48	0.92	0.83	0.36	0.79
$y2-H_2O$	-0.41	0.55	1.18	-0.28	1.12	0.86	0.38	0.73
y2'	-0.26	0.75	1.13	0.10	0.67	1.43	0.70	1.24

Table S2. Qualitative evaluation of the presence of detected metabolites in the extracts of *A. pachycristatus* strains.

ID	wt1	LaeA	veA	wt3	mcrA
Alternariol	+	+	+	+	+
Antibiotic 1656G	+	-	+	+	+
Antibiotic 3127	+	-	+	+	+
Aspercryptin A1	+	+	-	+	+
Aspercryptin A2	+	+	-	+	-
Aspernidine A	+	-	+	+	+
Aspernidine B	+	-	+	+	+
Aspernidine C	+	+	+	+	+
Emericellamide A	+	+	+	+	+
Emericellamide C/D	+	+	+	+	+
Emericellamide E	+	+	+	+	+
Emericellamide F	+	+	+	+	+
Emericellamide G	+	+	+	+	+
Emericellamide H	+	+	+	+	+
F9775-A	-	-	+	-	-
F9775-B	-	+	+	-	+
Fellutamide B	+	+	+	+	+
Fellutamide C	+	+	+	+	+
Fellutamide H	+	-	+	+	+
Fellutamide I	+	-	+	+	+
Fellutamide J	+	-	+	+	+
Fellutamide G	+	+	+	+	+
Sterigmatocystin	+	+	+	+	+
Triacetylfusarinine	+	+	+	-	+
Triacetylfusarinine +C_2H_4	-	-	+	-	+
Triacetylfusarinine +C3H6	-	-	+	-	-
Triacetylfusarinine +CH2	-	+	+	-	+

Table S3. Strains used in this study

Strain name	Genotype	Source
wt	Wild type (NRRL 11440)	NRRL
wt1	△Apc.pyrG△Apc.nkuA::pyr4	Lan et al., 2019
wt2	△Apc.pyrG△Apc.nkuA	Lan et al., 2019
wt3	△Apc.pyrG△Apc.nkuA△Apc.stcA::Af.pyrG	This study
wt4	△Apc.pyrG△Apc.nkuA△Apc.stcA	This study
△Apc.laeA	△Apc.laeA::pyr4△Apc.nkuA△Apc.pyrG	Lan et al., 2019
△Apc.veA	△Apc.veA::pyr4△Apc.nkuA△Apc.pyrG	Lan et al., 2019
△Apc.mcrA	△Apc.mcrA::Af.pyrG△Apc.nkuA△Apc.pyrG△Apc.stcA	This study

Table S4. Correspondence of secondary metabolic BGCs between strains *A. pachycristatus* NRRL 11440 and *A. nidulans* FGSC A4

Gene in NRRL 11440	Gene name in FGSC A4	Corresponding gene in FGSC A4	Product(s)	Type	Similarity %
g8962	_	AN10289	_	DMATS	86.8
g5595	nptA	AN11080	Nidulanin A	DMATS	92.9
g8074	xptA	AN6784	Variecoxanthone A	DMATS	89.9
g38	tdiB	AN8514	Terrequinone A	DMATS	97.1
g5798	_	AN0016	_	NRPS	92.2
g4770	sidD	AN6236	N',N'',N'''-triacetylfusarinine C	NRPS	95.6
g6534	sidC	AN0607	Ferricrocin	NRPS	91.1
g1578	_	AN10297	_	NRPS	90.3
g3816	_	AN10486	_	NRPS	92.4
g3916	ivoA	AN10576	N-acetyl-6-hydroxytryptophan	NRPS	92.5
g5244	nlsA	AN1242	Nidulanin A	NRPS	91.4
g3607	_	AN1680	_	NRPS	95.3
g9387	_	AN2064	_	NRPS	93.2
g5866	easA	AN2545	Emericellamide	NRPS	91.1
g4612	micA	AN3396	Microperfuranone	NRPS	93.3
g7876	inpA	AN3495	Fellutamide	NRPS	91.3
g7875	inpB	AN3496	Fellutamide	NRPS	92.2
g2105	_	AN4827	_	NRPS	95.1
g5330	_	AN5318	_	NRPS	97.9
g9187	_	AN6444	_	NRPS	91.4
g4410	_	AN6961	_	NRPS	63.5
g7715	_	AN7884	_	NRPS	92.1
g6236	_	AN8105	_	NRPS	89.5
g39	tdiA	AN8513	Terrequinone A	NRPS	94.1

g1387	_	AN9129	_	NRPS	83.9
g2105	_	AN4827	_	NRPS-like	88
g6893	mdpG	AN0150	Emodin/monodictyphenone	PKS	97.7
g3352	_	AN10430	_	PKS	88.5
g4405	_	AN1784	_	PKS	83.3
g5867	easB	AN2547	Emericellamide	PKS	93.7
g4218	pkfA	AN3230	Orsellinaldehydes	PKS	94.9
g4624	_	AN3386	_	PKS	92.1
g3354	_	AN3612	_	PKS	92.2
g143	aptA	AN6000	Asperthecin	PKS	97.7
g10105	_	AN6431	_	PKS	95.3
g9183	pkbA	AN6448	_	PKS	88.5
g5560	_	AN9005		PKS	96
g8067	_	AN6791	_	PKS	91.9
g5606	pkgA	AN7071	Alternariol/isocoumarins	PKS	79.2
g1929	_	AN7489	_	PKS	93.3
g7351	stcA	AN7825	Sterigmatocystin	PKS	93.9
g8717	_	AN7903	Violaceol I and II	PKS	93.5
g8711	orsA	AN7909	Orsellinic acid/F9975/violaceols	PKS	93.2
g7286	wA	AN8209	Green conidial pigment	PKS	96.7
g8765	_	AN8910	_	PKS	93.3
g8807	_	AN7838	_	PKS	86
g7276	_	AN6431	_	PKS	88
g3551	_	AN9217	_	PKS	90.9
g1312	_	AN2924	_	Acyl-CoA synthetase	83
g4204	ecdA	no hit	Echinocandin B	NRPS	0
g8810	_	no hit	_	NRPS	0
g9873	_	no hit	_	NRPS-like	0

g8115	_	no hit	_	NRPS-like	0
g1328	_	no hit	_	PKS	0
g1480	_	no hit	_	PKS	0
g6893	_	no hit	_	PKS	0
g7942	_	no hit	_	PKS	0
g2932	_	no hit	_	PKS	0
g8717	_	no hit	_	PKS	0
g9282	_	no hit	_	PKS	0
g9463	_	no hit	_	PKS	0
g9719	_	no hit	_	PKS	0
g9813	_	borderline hit	_	PKS	0
g9805	_	no hit	_	PKS	0
g1958	_	no hit	_	TS	0
g1957	_	no hit	_	TS	0
no hit	acvA/pcbAB	AN2621	Penicillin G	NRPS	0
no hit	_	AN2924	_	NRPS	0
no hit	asqK	AN9226	Aspoquinolone, Cyclopeptin	NRPS	0
no hit	_	AN9243	_	NRPS	0
no hit	_	AN9244	_	NRPS	0
no hit	_	AN9291	_	NRPS	0
no hit	adpA	AN8412	Aspyridone	PKS-NRPS	0
no hit	_	AN0523	_	PKS	0
no hit	afoE	AN1034	Asperfuranone	PKS	0
no hit	afoG	AN1036	Asperfuranone	PKS	0
no hit	_	AN11191	_	PKS	0
no hit	pkhA	AN2032	_	PKS	0
no hit	pkhB	AN2035	_	PKS	0
no hit	_	AN3273	_	PKS	0
no hit	_	AN5475	_	PKS	0

no hit	_	AN7084	_	PKS	0
no hit	ausA	AN8383	Austinol	PKS	0
no hit	_	AN1594	Ent-pimara-8(14),15-diene	TS	0
no hit	_	AN3252	_	TS	0
no hit	_	AN9314	_	TS	0
no hit	_	AN11202	_	DMATS	0
no hit	ausN	AN9259	Austinol, Dehydroaustinol	DMATS	0

Table S5. Primers used in this study.

Prime Name	Sequence (5′-3′)
P1-mcrA-ko-F1	CCCAAAACCTACATCTGGAC
P2-mcrA-ko-R1	GGCGTATCATTCAGAGCTGGTGGCGCAGACTTATCTCCACT
P3-mcrA-ko-F1	AGATAACAGCTTGGCATCACCAAAAAGCGGAAACTAAGGC
P4-mcrA-ko-R1	TCCAAATGCCTGATACTTGC
P#-A.f-pyrG-F1	TCACCCTCTTCGCGGG
P#-A.f-pyrG-R1	GTGATGCCAAGCTGTTATCT
P*-mcrA-ko-veri-up-F1	CCCAAAACCTACATCTGGAC
P*-veri-afpyG-up-R1	CAATCACTGGTAACTCCACG
P*-veri-afpyG-dw-F1	GAGCAAAAGTGTAGTGCCAG
P*-mcrA-ko-veri-dw-R1	ATGGTGATGAAACAGCGAGG
P1-stcA-ko-F1	AGGGACAGGTCATAGACTCG
P2-stcA-ko-R1	GACCCGCGAAGAGGGTGAGGAACTTATTGACCGCGTGA
P3-stcA-ko-F1	GAGATAACAGCTTGGCATCACTCGCCGAGTCAAGGATAGATG
P4-stcA-ko-R1	GTGATTCGCGAGTCGGATAG
P5-stcA-nested-F1	AATTCGTCCTGTTGGTATGC
P6-stcA-nested-R1	TTGAATCGGCTTCCACTTAC
P7*-stcA-veri-up-F1	CAGTTCCATATGCCGTTTG
P8*-stcA-veri-down-R1	GTATCCGAACGGGTTGAATG
P*-stcA-veri-F1	CTTCATCGATCACCTTGACC
P*-stcA-veri-R1	CCTGGTGAGTCTTGAAATGG
P1-pyrG-recyc-stcA-F1	AGGGACAGGTCATAGACTCG
P2-pyrG-recyc-stcA-R1	ATCTATCCTTGACTCGGCGAGGAACTTATTGACCGCGTGA
P3-pyrG-recyc-stcA-F1	TCACGCGGTCAATAAGTTCCTCGCCGAGTCAAGGATAGAT
P4-PyrG-recyc-stcA-R1	GTGATTCGCGAGTCGGATAG
